# Ultrasound in augmented reality: a mixed-methods evaluation of head-mounted displays in image-guided interventions

**DOI:** 10.1007/s11548-020-02236-6

**Published:** 2020-07-28

**Authors:** Christoph Rüger, Markus A. Feufel, Simon Moosburner, Christopher Özbek, Johann Pratschke, Igor M. Sauer

**Affiliations:** 1 Department of Surgery, Campus Charité Mitte | Campus Virchow-Klinikum, Experimental Surgery, Charité – Universitätsmedizin Berlin, Corporate Member of Freie Universität Berlin, Humboldt-Universität zu Berlin, and Berlin Institute of Health, Augustenburger Platz 1, 13353 Berlin, Germany; 2 Cluster of Excellence Matters of Activity, Image Space Material Funded by the Deutsche Forschungsgemeinschaft (DFG, German Research Foundation) Under Germany’s Excellence Strategy – EXC 2025, Augustenburger Platz 1, 13353 Berlin, Germany; 3 Scopis GmbH, Heinrich-Heine-Platz 10, 10179 Berlin, Germany; 4 Division of Ergonomics, Department of Psychology and Ergonomics (IPA), Technische Universität Berlin, Marchstr. 23, MAR 3-2, 10587 Berlin, Germany; 5 Technische Universität Berlin, Straße des 17. Juni 135, 10623 Berlin, Germany

**Keywords:** Augmented reality, Mixed reality, Extended reality, AR, MR, XR, HoloLens, Head-mounted display, Biopsy, Needle placement, Ultrasound, Image-guided, Ultrasound-guided, Ergonomics, Mixed methods, Evaluation, Usability, Human factors

## Abstract

**Purpose:**

Augmented reality (AR) and head-mounted displays (HMD) in medical practice are current research topics. A commonly proposed use case of AR-HMDs is to display data in image-guided interventions. Although technical feasibility has been thoroughly shown, effects of AR-HMDs on interventions are not yet well researched, hampering clinical applicability. Therefore, the goal of this study is to better understand the benefits and limitations of this technology in ultrasound-guided interventions.

**Methods:**

We used an AR-HMD system (based on the first-generation Microsoft *Hololens*) which overlays live ultrasound images spatially correctly at the location of the ultrasound transducer. We chose ultrasound-guided needle placements as a representative task for image-guided interventions. To examine the effects of the AR-HMD, we used mixed methods and conducted two studies in a lab setting: (1) In a randomized crossover study, we asked participants to place needles into a training model and evaluated task duration and accuracy with the AR-HMD as compared to the standard procedure without visual overlay and (2) in a qualitative study, we analyzed the user experience with AR-HMD using think-aloud protocols during ultrasound examinations and semi-structured interviews after the task.

**Results:**

Participants (*n* = 20) placed needles more accurately (mean error of 7.4 mm vs. 4.9 mm, *p *= 0.022) but not significantly faster (mean task duration of 74.4 s vs. 66.4 s, *p *= 0.211) with the AR-HMD. All participants in the *qualitative study* (*n *= 6) reported limitations of and unfamiliarity with the AR-HMD, yet all but one also clearly noted benefits and/or that they would like to test the technology in practice.

**Conclusion:**

We present additional, though still preliminary, evidence that AR-HMDs provide benefits in image-guided procedures. Our data also contribute insights into potential causes underlying the benefits, such as improved spatial perception. Still, more comprehensive studies are needed to ascertain benefits for clinical applications and to clarify mechanisms underlying these benefits.

**Electronic supplementary material:**

The online version of this article (10.1007/s11548-020-02236-6) contains supplementary material, which is available to authorized users.

## Introduction

Augmented reality head-mounted displays (AR-HMDs) have been envisioned as tools for visualizing image data in medical interventions since the mid-1990s [1–3]. Since then, technology has progressed significantly and technical feasibility has been shown in many studies [4–6]. Few studies, though, have systematically examined the effects of AR-HMDs on the tasks they aim to facilitate [7], particularly using established methods from psychological and human factors research.

*Intended benefits* of AR-HMDs are, for example, reduced workload, fewer complications or shorter interventions [6]. These benefits are directly relevant in clinical practice, but they are difficult to verify: Varying difficulty of cases and differences in practitioner skill have a large impact on outcomes and are difficult to control, particularly with small sample sizes. Furthermore, the intended benefits cannot be deduced directly from features of AR-HMDs. They are instead caused by *intermediate mechanisms*, such as improved eye-hand coordination, a better focus on the task, or a better spatial understanding of images and/or anatomy. These intermediate mechanisms, in turn, are supported by features inherent in the technology.^1^ For instance, AR may augment the view of a situs with additional contextual information; HMDs can provide additional depth cues through stereoscopic vision. We argue that the *intended benefits* of AR-HMDs should be seen as the *end of a chain of effects*—and that understanding the links in this chain is crucial to maximizing benefits in clinical interventions. In this study, we started to analyze this chain of effects, asking the question: *Do relevant benefits exist and if so, on which mechanisms are they based?*

We approached this question by examining the use of AR-HMDs in ultrasound-guided needle placements. We chose these placements as a representative image-guided task because of their relevance to a variety of use cases (e. g., biopsies, tumor ablation, and regional anesthesia) in numerous medical fields. In order to control external effects (such as interruptions and intervention difficulty) and to generalize results to a variety of possible use cases, the experiments were conducted in a lab setting and with simplified tasks.

To investigate potential benefits *and* underlying intermediate mechanisms, we used a mixed-methods approach, combining a quantitative experiment *and* a qualitative interview study. In the experiment, participants performed needle placements on a training model, using the AR-HMD or a standard ultrasound system in a random order. We compared placement error, task duration, and perceived workload as a measure of (intended) benefits. Additionally, we conducted a second, qualitative study using think-aloud protocols and semi-structured interviews with the main goal of identifying intermediate mechanisms. In contrast to a purely quantitative, outcome-focused approach, qualitative research is not limited to a set of a priori hypotheses and data. We think that this is particularly useful for illuminating issues that are not yet well researched and for which it is difficult to formulate well-founded hypotheses.

The experiment and the qualitative study were conducted at the same time and did not influence each other; their results were analyzed separately and combined in the general discussion section (following a convergent parallel design.) Accordingly, methods and results are reported in separate sections for each study after the presentation of the general technical setup.

To the best of our knowledge, the only other systematic evaluation of a comparable setup was conducted in 2001 with only a single participant [1]. Other evaluations either did not display images in 3D or did not utilize AR-HMDs. Most studies also focused on intended benefits without examining intermediate mechanisms and had rather small sample sizes (*n* < 10). Accordingly, our studies provide novel insights and expand the (still limited) body of evidence concerning practical clinical benefits of AR technology in image-guided interventions and their underlying mechanisms.

## General technical setup

**Fig. 1:**
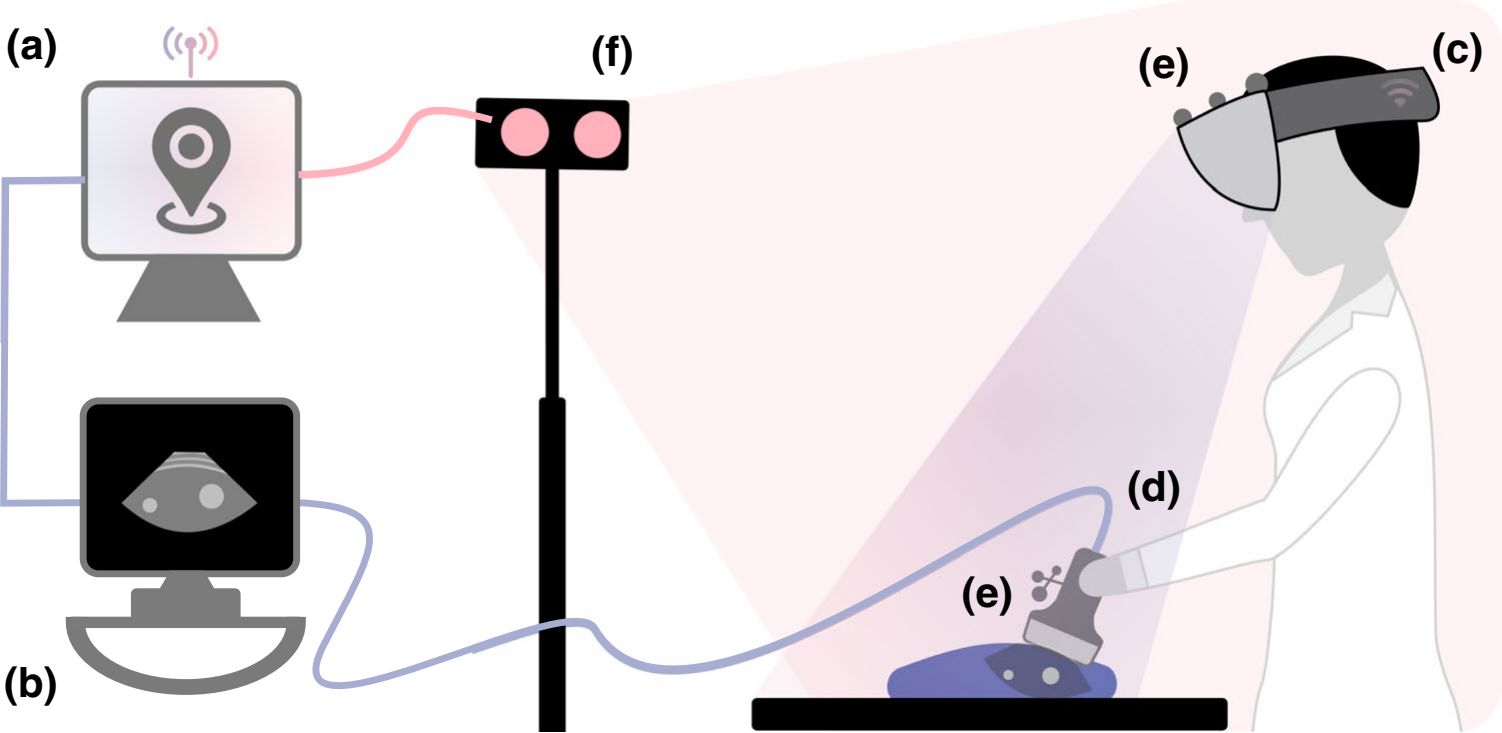
Illustration of the technical setup in use, consisting of **a** navigation system, **b** ultrasound system, **c** AR-HMD, **d** ultrasound transducer, **e** reflective markers, and **f** 3D camera

**Fig. 2:**
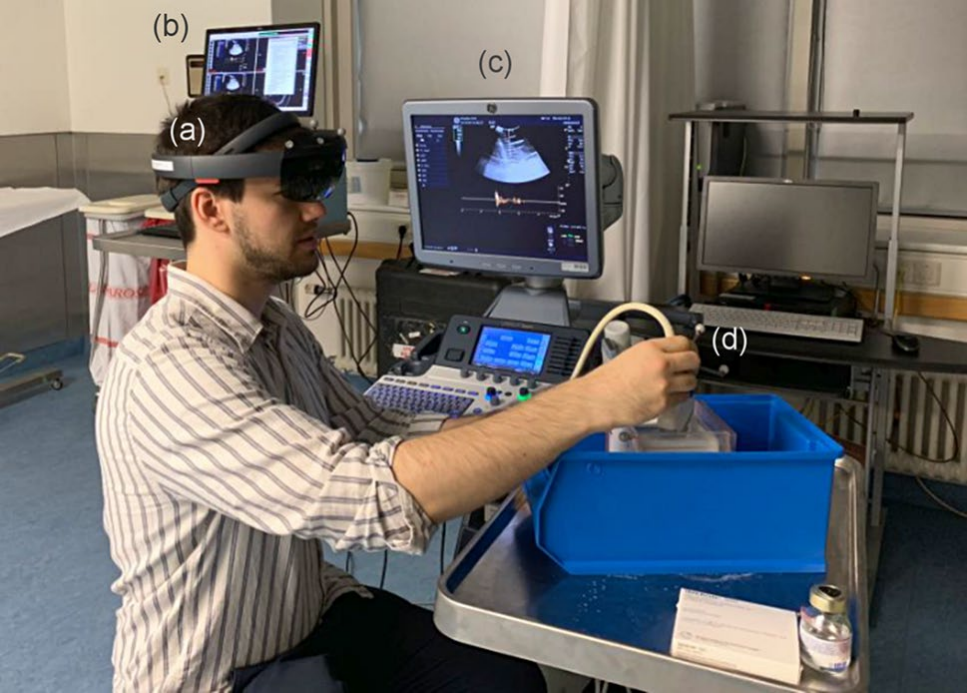
Exemplary use of **a** AR-HMD, **b** surgical navigation system, **c** ultrasound system, and **d** transducer

Both experiments were conducted in an outpatient interventional procedure room. The basic setup (Fig. 1) consisted of a surgical navigation system (Figs. 1a, 2b, Scopis GmbH, Berlin, Germany) and an ultrasound system (Figs. 1b, 2c, *Logiq S7*, GE Healthcare, Chicago, IL, USA), as well as an AR-HMD (Figs. 1c, 2a, *HoloLens (1st gen)*, Microsoft Corp., Redmond, WA, USA), using optical see-through technology. The choice regarding the AR-HMD in use was due to the *HoloLens* being the only commercially available stand-alone device that could render 3D content when software development on this research began (early 2018). The navigation system as well as the AR-HMD ran experimental proprietary software (by Scopis GmbH) modified for this study. Reflective markers (Fig. 1e) were attached to the AR-HMD and ultrasound transducer (Fig. 1d), which we then calibrated to enable stereoscopic optical tracking via the 3D camera (Fig. 1f). The initial calibration was part of the setup process and was handled via proprietary software from Scopis GmbH. The calibrations yielded the transformation matrices $_{UStracker} T^{USimage}$ and $_{HMD} T^{HMDtracker}$, which described the position and rotation of the ultrasound image relative to the tracker on the ultrasound transducer, respectively, of the HMD tracker relative to the origin of the AR-HMD (which was used for generating the graphics output).

The navigation system grabbed the screen output of the ultrasound system via its secondary HDMI output port. It then trimmed out the ultrasound system’s user interface so that only the actual ultrasound image (B-mode) remained, with a resulting resolution of approximately 600 by 600 pixels. Utilizing the calibration data from the setup and the real-time tracking data from the 3D camera, the transformation matrix of the ultrasound image relative to the HMD tracker ($_{HMD} T^{USimage}$) was then calculated:

$$ _{HMD} T^{USimage} = _{HMD} T^{HMDtracker} \cdot_{HMDtracker} T^{3Dcamera} \cdot _{3Dcamera} T^{UStracker} \cdot _{UStracker} T^{USimage} $$

The navigation system then sent the trimmed ultrasound image and the corresponding spatial data ($_{HMD} T^{USimage}$) to the HMD via a TCP/IP socket in a wireless local network, at a frequency of 10 frames per second. Upon receiving the data, the HMD software rendered the received image at the ultrasound transducer (as encoded in $_{HMD} T^{USimage}$).

The process outlined above cannot account for the position of the HMD relative to the user’s eyes which may change every time the HMD is put on. This leads to a reduced overlay accuracy. To address this issue, the HMD software included an additional user calibration, which was to be run every time the HMD was put on. The calibration process employed the single-point active alignment method [8], where users aligned a virtual point to spheres of a 3D tracker. At the end of the calibration process, the spheres were virtually displayed so that users could judge the accuracy of the overlay by comparing the distance between the virtual spheres and the spheres of the real tracker. With this additional calibration step, we could achieve an overlay accuracy of roughly 5 mm.

A similar setup was described in more detail by *Kuzhagaliyev* et al. [9]. Our system additionally included the user calibration step described above, but did not feature a needle alignment guide along predefined paths.

## Experimental study (needle placement)

The goal of the experiment was to assess intended benefits of an AR-HMD system in a representative image-guided task. Other AR-HMD setups have been shown to provide performance benefits [10–13]—therefore, we formulated our primary hypotheses as an attempt to replicate these results: AR-HMDs allow participants to place needles *faster* and *more accurately*.

Our secondary hypotheses were directed toward causes of the benefits. We assumed that those with *limited ultrasound experience* were less skilled at transferring and imagining 2D ultrasound images in 3D. We postulated that this process would impact needle placement performance. We also expected that the HMD would facilitate this 2D-to-3D transformation—making the skill (deficit) less important and allowing beginners to perform better. Accordingly, our secondary hypotheses were that *participants who had performed fewer examinations/procedures would benefit more in terms of task duration, placement error and subjective task load* from using the AR-HMD, as it displays the images in 3D space and removes the need to translate 2D into 3D information. Another secondary hypothesis was that the perceived task load would be lower when using the AR-HMD, independent of ultrasound experience.

## Methods

We recruited participants (*n *= 20) with different skill levels through mailing lists of the surgical department and in person. Requirements for participation were (1) basic ultrasound skills, (2) proficiency in English or German, and (3) no previous experience with AR-HMDs. We sampled by clinical experience (to reflect different skill levels) as well as by convenience due to limited availability of the equipment. Of the 20 participants, 7 were female, 13 were male. Thirteen participants were medical students, 7 physicians. Ultrasound experience ranged from 3 to 650 procedures/examinations, with a mean of 156.2 and a median of 35.

We tested our hypotheses by comparing performance differences in ultrasound-guided needle placements. The experiment used a crossover design, where participants either started in the standard or AR-HMD condition. The order of the conditions was randomized through coin tosses to control for learning and carry-over effects. We measured placement error and task duration; perceived task load was assessed through the NASA task load index (TLX) questionnaire [14]. We also noted the approximate number of ultrasound examinations and ultrasound-guided interventions each participant had performed (based on self-report) as a measure of experience.

The basic technical setup was expanded with a tracked biopsy training model (*Blue Phantom* soft tissue biopsy ultrasound model, CAE Healthcare, Sarasota, USA) and a tracked biopsy needle (Quick-Core, 18ga., 15 cm—Cook Medical; Bloomington, USA). An MRI scan (TSE T2, 0.8 mm slice thickness) of the model and the tip of the biopsy needle were registered to the respective trackers. The model contained 16 echogenic masses (diameters 4–11 mm, distance between centers approx. 30–40 mm); their centers were labeled in the MRI dataset in the navigation system.

**Fig. 3:**
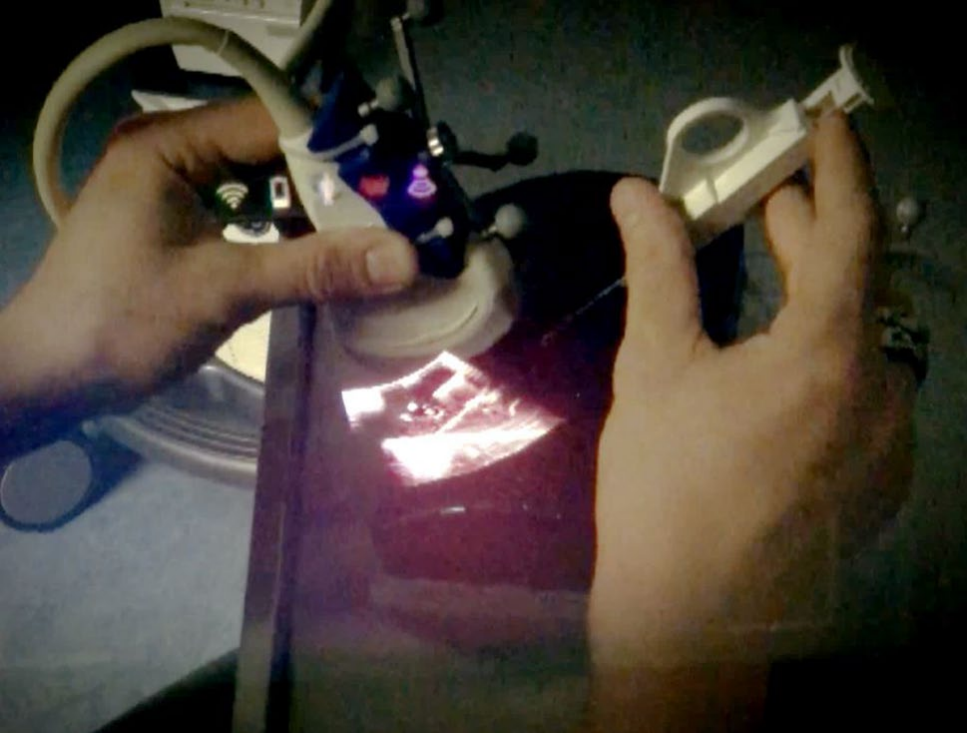
Ultrasound-guided needle placement with image overlay at the transducer (image was captured through the HMD resulting in limited resolution; the actual overlay accuracy was higher)

Participants sat in front of a table with the setup of the biopsy model described above. They began the task in a ‘ready’ pose, holding the ultrasound transducer and biopsy needle above the model. They were then signaled to begin, and a stopwatch was started. After locating a mass of their choosing and determining its center, participants placed the needle (Fig. 3). They could then slightly adjust the position of the tip, but not completely retract and re-insert, that is, one insertion counted as one attempt. The stopwatch was stopped to measure task duration when participants signaled that they had finished adjustments. The distance between the tracked needle tip and the nearest mass center (placement error) was then measured with the navigation system. Participants performed the task twice per condition, i.e., twice with and twice without the AR-HMD, for a total of four needle placements. After each condition, participants electronically answered the TLX questionnaire (that is, two times in total). A video of an exemplary needle placement attempt is available in Online Resource 1.

We conducted statistical analyses with RStudio (version 1.1.463, R version 3.6.1, code attached in Online Resource 6), using Wilcoxon signed-rank tests for our primary hypotheses (reduced placement error and task duration with AR-HMD) as well as one secondary hypothesis (reduced task load in the AR-HMD versus standard condition for less experienced participants). Kendall’s $\tau_{a}$ was calculated as a measure of correlation between performance differences and the number of previously performed examinations/procedures (secondary hypotheses).

## Results

We calculated performance differences as specified below. Accordingly, *positive differences* show that a participant performed *better with the HMD,* because a shorter task duration, lower placement error and task load are preferable.

$$ \Delta x = x_{\text{control}} - x_{\text{HMD}} $$

**Fig. 4:**
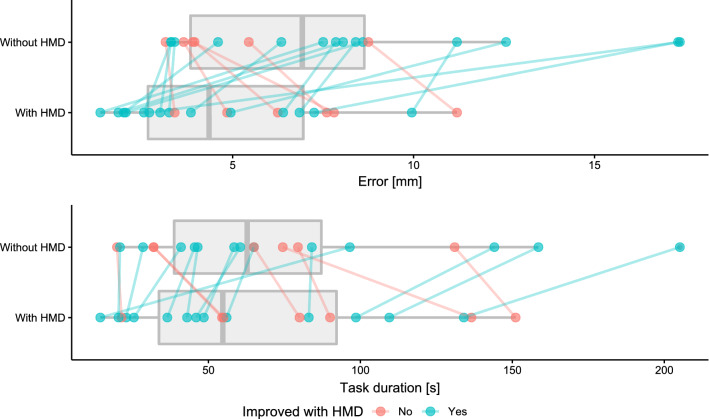
Box-plots and dot-plots showing averaged task duration and placement error; lines connect the data points that belong to an individual participant

With the HMD, participants (*n* = 20) placed needles more accurately (mean error *μ* = 5.0 mm standard deviation *σ* = 2.8 mm vs. *μ* = 7.4 mm, *σ* = 4.4 mm without HMD; *p* = 0.022 with *α* = 0.025 after Bonferroni correction, Fig. (4). The reduction in task duration was not statistically significant (*p* = 0.211) between the HMD condition (*μ* = 66.4 s, *σ* = 42.0 s) and the control condition (*μ* = 74.4 s, *σ* = 50.1).

**Fig. 5:**
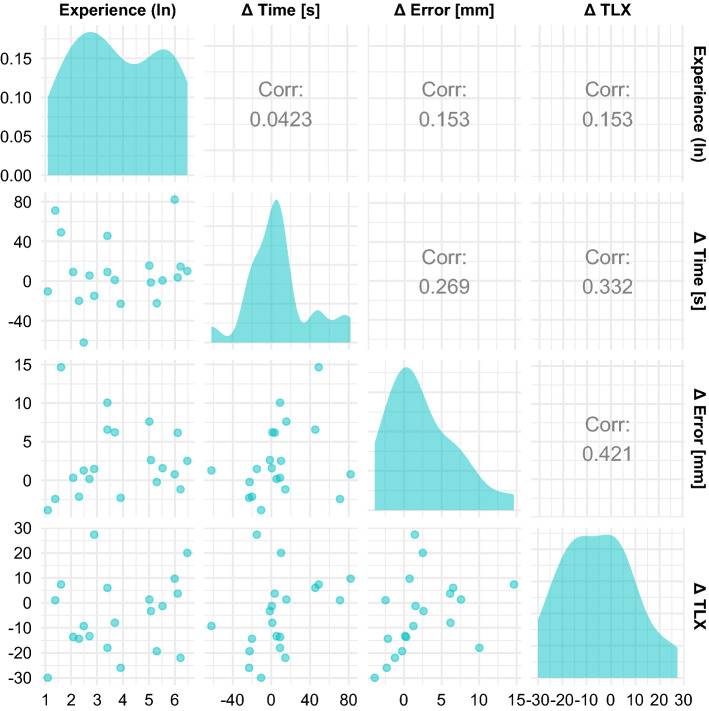
Table of scatter plots (lower left) and Kendall’s $\tau_{a}$ showing correlation between measured variables (upper right); density plots (diagonal) visualize distributions

We rejected our secondary hypotheses: (1) Task load was not reduced (mean TLX score of *μ* = 43.5 for the standard setup vs. *μ* = 48.6 with HMD, *p* = 0.147) and (2) experienced participants did not have a smaller performance difference or benefit (i.e., a negative correlation between performance differences and ultrasound experience; $\tau_{a}$ = 0.0423 for differences in task duration, $\tau_{a}$ = 0.153 for differences in error, Fig. 5). However, exploratory data analysis reveals moderate correlations between differences in TLX scores and performance differences (Fig. 5); differences in task duration and error also seem weakly to moderately correlated in the sample (Spearman’s $\rho$  = 0.33).

The order of the conditions appeared to have a strong impact on the differences in task duration: Participants were roughly 30 s faster when using the AR-HMD if this was not their starting condition. If they performed their first two placements with the AR-HMD, however, they were on average 10.5 s slower when doing so. Of the 20 participants, 11 performed the first two needle placements with the AR-HMD. All measurements are available in a tabular format in Online Resource 7 (Fig. 5).

## Qualitative study (ultrasound examination)

The biopsy model we used for the experiment above (needle placements) is abstract; it does not contain any echogenic features besides the round biopsy targets. However, a common challenge in image-guided interventions is to mentally translate anatomical structures from 2D images into a 3D representation, for example in order to align a tool in 3D space.^2^ Because anatomical structures are more complex than the biopsy training model, we consider it important to also examine the effects of the AR-HMD system when used in the context of human anatomy. As it is difficult to reliably quantify anatomical understanding, this study focused on user experience and subjective remarks on anatomical perception and understanding.

The study relates to the research question in two ways: In addition to quantitatively measured benefits, we were also interested in subjective benefits as perceived by practitioners. While not easily generalizable, such subjective impressions might be the basis for new hypotheses and use cases to be examined in the future. Additionally, we hoped to gain insights to the second part of our research question: Finding intermediate mechanisms that enable intended benefits like accuracy improvements. Accordingly, we attempted to identify differences in the way participants handle the task and how they perceive ultrasound images. The experiment was conducted in German; categories and quotes by participants cited in the manuscript have been translated by the authors.

## Methods

We followed the same recruitment procedure as in the quantitative experiment. However, participants were required to be comfortable performing abdominal ultrasound examinations, rather than just possessing basic skills. The experiment included 6 participants: 2 residents and 2 senior physicians of the department of general surgery as well as 2 students tutoring sonography courses; 5 participants were male.

**Fig. 6:**
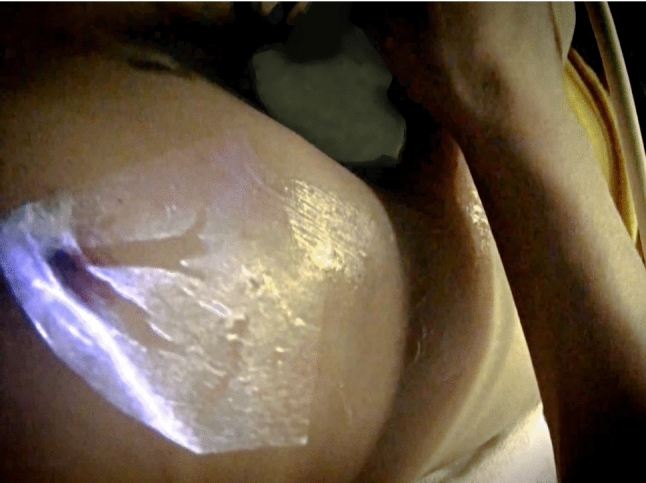
Ultrasound image overlaid onto the abdomen, captured through the HMD

Participants performed abdominal ultrasound examinations on two volunteers, once using the standard procedure and once using the AR-HMD (Fig. 6, see Online Resource 2 for a video). During both examinations, participants were asked to verbalize their thoughts (think-aloud protocol [15]). Afterward, they participated in a semi-structured interview (see Online Resource 4 for the interview guideline).

After informed consent and instructions, participants were asked to think aloud while performing the ultrasound examination on the first subject, using the monitor of the standard ultrasound system. To allow them to adjust to the study setting, participants *always* started with the standard system. By not introducing the AR-HMD at first, participants could mentally transition to performing ultrasound examinations after interrupting their regular clinical routine. For the second examination, participants put on the AR-HMD, which overlaid the ultrasound image at the transducer. While we asked participants to focus on the AR-HMD image, participants were free to also look at the monitor. After the second examination, we concluded sessions with a short break and a one-on-one semi-structured interview. Additionally, field notes were taken throughout the sessions to record general observations and how participants used ultrasound imaging in their work.

The first author conducted these studies, transcribed the recorded audio and analyzed the data. The goal of the analysis was to identify benefits and limitations perceived by the participants, as well as changes in perception and task execution compared to the standard procedure. The transcriptions were examined through qualitative content analysis as described by Kuckartz [16]. In this process, the text was reduced to relevant passages (so- called codes), which were then assigned to categories. The categories were generated both deductively from theory and inductively from codes. Additionally, think-aloud protocols and interviews were summarized for each participant to obtain cohesive overviews. To improve rigor, we employed (1) method triangulation, combining the strengths of think-aloud protocols and interviews, (2) iterative generation and assignment of categories, and (3) comparisons between extreme (deviant) cases.

## Results

We identified six main categories relevant to the research question (Table 1, for category definitions, see Online Resource 5). With 25–35% of all passages coded per participant, *limitations* is the most frequent category. Its most common subcategories were ‘*view depends on angle’* (image is not visible when ultrasound plane is parallel to view direction) and ‘*ergonomics’* (physical discomfort like neck pain.) All participants verbalized unfamiliarity with this technology (category ‘*Familiarity’*), often mentioning that unfamiliarity made it difficult to use the technology. However, all but one participant expected that unfamiliarity could be mitigated by training. Subcategories of ‘*benefits’* are ‘*easier navigation’* (within the patient anatomy), ‘*improved 3D perception’* (of anatomical structures), and ‘*hedonistic aspects’* (e.g., novelty); the latter was the most common benefit (50% of all *benefits* coded).

**Table 1:** Distribution of categorized codes per participant

	P1	P2	P3	P4	P5	P6	Total
Limitations	19	17	7	8	14	13	78
Familiarity	17	2	8	7	13	13	36
Benefits	10	4	3	7	11	1	10
Feasibility	3	1	2	2	1	1	60
Perception	4	7	2	3	1	4	21
Assessments	5	14	7	2	5	11	44
Total	71	61	46	44	58	60	

*Feasibility* includes positive remarks about *image quality* and *ergonomic aspects*. Multiple participants remarked that tolerating discomfort in the OR is common—and that ergonomic issues of the HMD appear acceptable in perspective. *Perception* includes statements about the sensory input used by participants to complete the task; most participants reported they did not notice a difference in sensory input when using the AR-HMD, particularly regarding the proprioceptive handling of the ultrasound probe. Codes assigned to *assessments* include verdicts about the usefulness of the technology and ideas for future use cases. Suggested use cases generally involved precise spatial alignment or targeting. Similarly, multiple participants expected that AR-HMDs would be used in complex cases, rather than routine cases.

Despite the small sample size, there were strong contrasts between participants, particularly between participant 2 (*P2*) and P1, P4, and P5. P2, a resident, dismissed the basic approach of using an AR-HMD for this task, even if it was ‘exciting’: P2 was used to imagining ultrasound images as ‘anatomical slices’ and thus found displaying images in 3D on the patient to be redundant; at the same time, the AR-HMD introduced new issues such as angle-dependent views. In contrast, P5, also a resident, saw ‘clear benefits,’ finding it easier to understand the patient anatomy, mentioning they could mentally draw in the resection line [if this were a partial hepatectomy].

Participants seemed to have varying techniques of processing ultrasound images. Two participants explicitly stated they had no issues imagining ultrasound images in 3D. Of the two, one was dismissive toward the AR-HMD (P2) and the other was only mildly positive (P6). A similar difference was mentioned by P6, an ultrasound tutor, which they observed throughout the ultrasound courses they held: Some students found it difficult to look at and interpret the ultrasound image while simultaneously orienting the transducer (instead of looking at the transducer), whereas other do not. Their half-joking explanation for this observation was that those who struggle likely did not play video games when they were younger.

## General discussion

Our results provide partial, though still incomplete, answers to the research question: *Do relevant benefits exist and if so, on which mechanisms are they based?* The AR-HMD provided quantifiable as well as subjective benefits; we could not verify all predicted benefits, though. Through qualitative data analysis, we were able to outline that improvements to spatial perception and understanding may be important intermediate mechanisms and be subject to individual differences. Even though the qualitative study design does not allow for causal inference, these insights may be foundations for hypotheses to be examined in future studies.

Participants were significantly more accurate when using the AR-HMD, reducing the mean error by a third (7.4 mm vs. 5.0 mm, *p* = 0.022). While we see this as further evidence for benefits of AR-HMDs, our results are not entirely conclusive: Task duration and perceived task load were both not significantly reduced. These inconclusive results may be due to unfamiliarity with the new technology, which has played an unexpectedly large role in the qualitative study. Unfamiliarity could lead to a higher task load (as mentioned in the qualitative study) and slower execution time—and possibly explain why task load and duration were not significantly reduced. The influence of unfamiliarity on the needle placement experiment is also supported (if anecdotally) by remarks of participants, recorded in field notes. Additionally, the strong effect of the order of conditions (i.e., starting with or without HMD) possibly obscured the effect we hypothesized. But despite these mixed results, we argue that an underlying effect exists: In addition to improved accuracy, exploratory data analysis shows moderate correlations between differences in task load and differences in accuracy and task duration; participants who found the task easier with the AR-HMD also tended to perform better.

Results of the qualitative study also reflected these ambivalent findings. All participants mentioned limitations, yet 5 out of 6 participants stated clear benefits and/or would like to test this technology in practice. Multiple participants verbalized improvements in 3D perception and navigation within the patient’s anatomy. However, one participant did not see any added value whatsoever. The strong contrast in judgments may be attributed to the participants’ primary field of experience: Those with a more diagnostic background seemed reluctantly favorable to dismissive; those with a focus on interventional ultrasound were in favor of the technology. An explanation for this may be that diagnostic ultrasound examinations generally revolve around measuring key parameters from 2D images—in interventions, however, it is crucial to understand the patient anatomy in 3D in order to align and navigate tools, for example.

In summary, we consider our combined qualitative and quantitative findings to further support current tendencies in the literature [10–13]: HMDs can offer benefits in image-guided interventions. However, whether these benefits can be practically realized in clinical settings requires further research.

Our data point to multiple potential causes for such benefits. Four out of six participants (in the qualitative study) mentioned an improvement of their spatial understanding of the ultrasound images. They stated that they could better locate the image in the patient anatomy and/or three-dimensionally imagine the anatomical structures they examined. Additionally, participants often mentioned use cases that require precise spatial navigation which implies benefits in such scenarios. Accordingly, we suggest *improved 3D*-*perception and navigation* as likely candidates for intermediate mechanism that may lead to practical benefits. These mechanisms may be relevant not only in ultrasound-related interventions but generally in image-guided procedures. However, individual differences in 3D perception and visuomotorics might make such effects less pronounced or redundant (as seen with P2 in our qualitative study).

Several participants stated they would rather use the AR-HMD in complex scenarios than for routine cases. On one hand, this could mean that they perceived the technology as a burden that should be used sparingly. On the other hand, they might feel that AR-HMDs augment their existing skills—which would only be relevant in cases they cannot handle proficiently already. If that is the case, AR-HMDs could help close the skill gap between novice and experienced surgeons or guide procedures that are only performed infrequently at a clinic. Accordingly, we suggest examining the relationship between the difficulty of a case and measurable benefits of the technology in future studies.

Hedonistic aspects were frequently mentioned in the qualitative study; that is, the technology was perceived as novel and/or exciting. Though it may temporarily improve user experience, we doubt that novelty offers major clinical benefits. It may even skew perception and inflate expectations of HMDs—both for study participants and in scientific debate.

Our a priori assumption about underlying causes did not hold: Less experienced participants (i.e., those with a lower number of ultrasound-guided interventions/examinations) *did not* seem to benefit more from using AR-HMDs. Instead, exploratory analysis shows that this number of ultrasound-guided tasks does not even correlate with placement performance when using the conventional ultrasound system. Accordingly, we consider it to be an ineffective predictor for beneficial effects of AR-HMD in this task and possibly for ultrasound-based needle placement performance in general.

Given the ambivalence in participants’ responses, we conclude that intermediate mechanisms seem to vary among users. The qualitative study suggests that some may not benefit from the AR-HMD technology, depending on, for instance, individual differences in 3D perception or visuomotorics. Additionally, benefits may only be noticeable during certain types of tasks and/or difficulty levels of interventions rather than in routine tasks. In conclusion, improved spatial understanding (through various pathways) seems to be a likely cause for the benefits of AR-HMDs. Based on these insights, we suggest that future research into the benefits of AR-HMD should investigate the intermediate mechanisms underlying the spatial understanding of ultrasound imaging and their interaction with person and task variables.

### Limitations of this study

Limitations of our study can be divided into three main categories: sampling, study design, and unfamiliarity. Both the quantitative experiment and the qualitative study have limitations related to the sampling of participants. Sample sizes were small because we were limited by the availability of the technical equipment. We also had to stop the needle placement experiment early because the training model had accumulated too much damage—previous needle cuts were clearly visible in the ultrasound image and distorted experimental conditions for later participants (Online Resource 3). Furthermore, our sampling was skewed in the following ways: (1) Participants were arguably inclined to be in favor of HMDs, considering they agreed to volunteer, (2) females were underrepresented, and (3) students were overrepresented in the quantitative experiment.

In addition to sampling issues, generalizability of our results is limited due to study design. Though we would like to draw conclusions for image-guided procedures in general, only ultrasound was used for imaging and we examined just two (relatively easy) tasks. Furthermore, we suspect that the accuracy of error measurements was negatively impacted by inhomogeneity in the MRI dataset and by participants accidentally bending the needle during measurements. We tried to mitigate these issues by averaging two measurements per condition per participant. Additionally, it is possible that overlay inaccuracies may have negatively impacted needle placement accuracy when using the AR-HMD. Finally, the impact of the order of the conditions (on the differences in task durations at least) implies that participants should perform ‘test runs’ before the initial measurement. Though we did consider this, we decided against it as we suspected limited durability of the biopsy model.

We initially considered no familiarity with HMDs to be beneficial for controlling variance. However, displaying ultrasound images in their ‘spatially correct’ position is not per se intuitive. Instead, participants generally found this way of displaying images to be unfamiliar or even strange. Though some seemed to adjust quickly during the experiments, multiple participants mentioned that lack of familiarity made the AR-HMD difficult to use in the qualitative study. We consider it likely that this effect was also present in the quantitative experiment. Generally, comparing familiar technology (conventional ultrasound) against a novel system (AR-HMD) may be considered biased for similar reasons: Adapting to novel situations generally results in (additional) cognitive load, making it harder to perform other tasks simultaneously. Furthermore, familiarity with a technology may have given users the opportunity to find strategies/techniques to better utilize benefits and mitigate limitations of the technology. As a result, we recommend effects of unfamiliarity to be sufficiently considered in the design of future studies.

## Conclusion

Our results add to the existing evidence that AR-HMDs can help improve performance in image-guided interventions. We additionally provide new evidence concerning potential intermediate mechanisms underlying these benefits. In particular, we suggest that the benefits of AR-HMDs are (at least partially) mediated by improvements of the spatial understanding of (ultrasound) images. Potential mechanisms are that transferring 2D images into mental 3D representations of the anatomy and/or estimating the position of the image in 3D is facilitated.

Based on our initial insights, we suggest various implications for future research, ideally to be performed in multicenter studies: Our participants consistently found the concept (and usage) of AR-HMDs to be unfamiliar, even strange. As a result, we recommend mitigating unfamiliarity by allowing participants to practice extensively before starting the actual experiment. Furthermore, we found *mixed methods* (combining quantitative and qualitative data) to be useful for interpreting results and providing a more differentiated perspective on study results, in particular at early stages of research. For instance, based on statements from the qualitative study, AR-HMDs may be particularly useful in difficult scenarios. Future studies could validate whether there is indeed a correlation between task difficulty and the magnitude of benefits; such a correlation would be important for identifying good clinical use cases.

We also reaffirm that *intermediate mechanisms*, rather than direct clinical benefits, should be the focus of upcoming studies in this field. Ultimately, we think that the current goal should be to determine the *causal pathways* underlying the clinical benefits of the technology—which skills and processes can it facilitate and how? By leveraging such knowledge, we believe that AR-HMDs can soon be applied and translated into clinical use cases with maximum impact.

## Electronic supplementary material

Below is the link to the electronic supplementary material.

Online Resource 1: Video of needle placement, filmed through the AR-HMD (MP4 4827 kb)

Online Resource 2: Video of ultrasound examination of a test patient, filmed through the AR-HMD (MP4 10760 kb)

Online Resource 3: Example image of ‘used-up’ model (PNG 246 kb)

Online Resource 4: Interview guideline (PDF 277 kb)

Online Resource 5: Category definition (categories of content analysis) (PDF 109 kb)

Online Resource 6: R code used for data analysis and plotting (R 9 kb)

Online Resource 7: Quantitative Data (PDF 667 kb)

## Footnotes

1 We refer to the concept of *affordances*, a concept introduced by James Gibson and later popularized by Don Norman to refer to the functions a technology affords its users.

2 This observation reflects our focus on surgery—we are aware that some radiologists may disagree with calling this task a challenge.

## References

[1] Rosenthal M, State A, Lee J, Hirota G, Ackerman J, Keller K, Pisano ED, Jiroutek M, Muller K, Fuchs H (2001) Augmented reality guidance for needle biopsies : a randomized, controlled trial in phantoms, pp 240–248 10.1016/s1361-8415(02)00088-9 12270235

[2] Peuchot B, Tanguy A, Eude M (1995) Virtual reality as an operative tool during scoliosis surgery. In: Ayache N (ed) Computer vision, virtual reality and robotics in medicine: first international conference, CVRMed’95, Nice, France, April 3–6, 1995 Proceedings, Springer, Berlin, pp 549–554

[3] Fuchs H, Livingston MA, Raskar R, Colucci D, Keller K, State A, Crawford JR, Rademacher P, Drake SH, Meyer AA (1998) Augmented reality visualization for laparoscopic surgery, pp 934–943. 10.1007/BFb0056282

[4] Badiali G, Ferrari V, Cutolo F, Freschi C, Caramella D, Bianchi A, Marchetti C (2014). Augmented reality as an aid in maxillofacial surgery: validation of a wearable system allowing maxillary repositioning. J Cranio Maxillofac Surg.

[5] Sauer IM, Queisner M, Tang P, Moosburner S, Hoepfner O, Horner R, Lohmann R, Pratschke J (2017). Mixed reality in visceral surgery. Ann Surg.

[6] Vávra P, Roman J, Zonča P, Ihnát P, Němec M, Kumar J, Habib N, El-Gendi A (2017). Recent development of augmented reality in surgery: a review. J Healthc Eng.

[7] Dey A, Billinghurst M, Lindeman RW, Swan JE (2018). A systematic review of 10 years of augmented reality usability studies: 2005–2014. Front Robot AI.

[8] Tuceryan M, Navab N (2000) Single point active alignment method (SPAAM) for optical see-through HMD calibration for AR. In: Proceedings of IEEE ACM international symposium augment reality, ISAR 2000, pp 149–158. https://doi.org/10.1109/ISAR.2000.880938

[9] Kuzhagaliyev T, Janatka M, Vasconcelos F, Clancy NT, Clarkson MJ, Hawkes DJ, Gurusamy K, Davidson B, Stoyanov D, Tchaka K (2018) Augmented reality needle ablation guidance tool for irreversible electroporation in the pancreas. In: Webster RJ, Fei B (eds) Medical imaging 2018: image-guided procedures, robotic interventions, and modeling, SPIE, p 30

[10] Przkora R, Mcgrady W, Vasilopoulos T, Gravenstein N (2015) Evaluation of the head-mounted display for ultrasound-guided peripheral nerve blocks in simulated regional anesthesia, pp 2192–2194 10.1111/pme.12765 25930716

[11] Al Janabi HF, Aydin A, Palaneer S, Macchione N, Al-Jabir A, Khan MS, Dasgupta P, Ahmed K (2019). Effectiveness of the HoloLens mixed-reality headset in minimally invasive surgery: a simulation-based feasibility study. Surg Endosc.

[12] Patrzyk M, Klee M, Stefaniak T, Heidecke CD, Beyer K (2018). Randomized study of the influence of two-dimensional versus three-dimensional imaging using a novel 3D head-mounted display (HMS-3000MT) on performance of laparoscopic inguinal hernia repair. Surg Endosc.

[13] Pelanis E, Kumar RP, Aghayan DL, Palomar R, Fretland ÅA, Brun H, Elle OJ, Edwin B (2019). Use of mixed reality for improved spatial understanding of liver anatomy. Minim Invasive Ther Allied Technol.

[14] Hart SG, Staveland LE (1988). Development of NASA-TLX (Task Load Index): results of empirical and theoretical research. Adv Psychol.

[15] Ericsson KA, Simon HA (1980). Verbal reports as data. Psychol Rev.

[16] Kuckartz U (2018) Qualitative Inhaltsanalyse. Methoden, Praxis, Computerunterstützung, 4th edn. Beltz, Weinheim

