# Correction to: Ultrasound in augmented reality: a mixed-methods evaluation of head-mounted displays in image-guided interventions

**DOI:** 10.1007/s11548-021-02467-1

**Published:** 2021-08-03

**Authors:** Christoph Rüger, Markus A. Feufel, Simon Moosburner, Christopher Özbek, Johann Pratschke, Igor M. Sauer

**Affiliations:** 1 Department of Surgery, Campus Charité Mitte | Campus Virchow-Klinikum, Experimental Surgery, Charité – Universitätsmedizin Berlin, Corporate Member of Freie Universität Berlin, Humboldt-Universität zu Berlin, Berlin Institute of Health, Augustenburger Platz 1, 13353 Berlin, Germany; 2 Cluster of Excellence Matters of Activity, Image Space Material Funded by the Deutsche Forschungsgemeinschaft (DFG, German Research Foundation) Under Germany´s Excellence Strategy – EXC, Augustenburger Platz 1, 13353 Berlin, Germany; 3 Scopis GmbH, Heinrich-Heine-Platz 10, 10179 Berlin, Germany; 4 Division of Ergonomics, Department of Psychology and Ergonomics (IPA), Technische Universität Berlin, Marchstr. 23, MAR 3-2, 10587 Berlin, Germany; 5 Technische Universität Berlin, Straße des 17. Juni 135, 10623 Berlin, Germany

## Correction to: International Journal of Computer Assisted Radiology and Surgery (2020) 15:1895–1905 10.1007/s11548-020-02236-6

The article Ultrasound in augmented reality: a mixed‑methods evaluation of head‑mounted displays in image‑guided interventions, written by Christoph Rüger, Markus A. Feufel, Simon Moosburner, Christopher Özbek, Johann Pratschke and Igor M. Sauer, was originally published Online First without Open Access. After publication in volume 15, issue 11, page 1895–1905 the author decided to opt for Open Choice and to make the article an Open Access publication. Therefore, the copyright of the article has been changed to © The Author(s) 2021 and this article is licensed under a Creative Commons Attribution 4.0 International License, which permits use, sharing, adaptation, distribution and reproduction in any medium or format, as long as you give appropriate credit to the original author(s) and the source, provide a link to the Creative Commons licence, and indicate if changes were made. The images or other third party material in this article are included in the article’s Creative Commons licence, unless indicated otherwise in a credit line to the material. If material is not included in the article’s Creative Commons licence and your intended use is not permitted by statutory regulation or exceeds the permitted use, you will need to obtain permission directly from the copyright holder. To view a copy of this licence, visit http://creativecommons.org/licenses/by/4.0/.

Open Access funding enabled and organized by Projekt DEAL.

The original article has been corrected.

