# Supplementary figures and images for: Ultrasound in augmented reality: a mixed-methods evaluation of head-mounted displays in image-guided interventions
Source: Int J Comput Assist Radiol Surg. 2020 Jul 28;15(11):1895–905. doi: 10.1007/s11548-020-02236-6 (PMC8332636; doi:10.1007/s11548-020-02236-6)

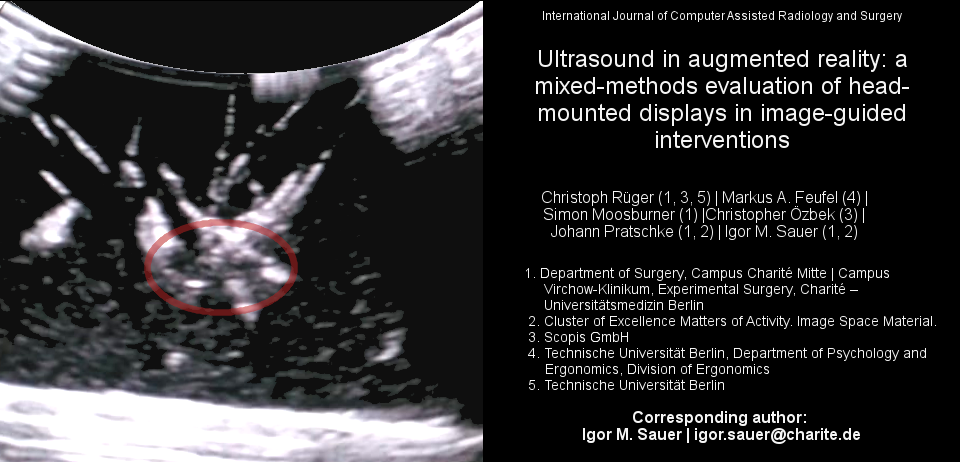

Supplement: Supplementary file 3 — Online Resource 3: Example image of ‘used-up’ model (PNG 246 kb) [file 11548_2020_2236_MOESM3_ESM.png]
