# Supplementary material for: Ultrasound in augmented reality: a mixed-methods evaluation of head-mounted displays in image-guided interventions
Source: Int J Comput Assist Radiol Surg. 2020 Jul 28;15(11):1895–905. doi: 10.1007/s11548-020-02236-6 (PMC8332636; doi:10.1007/s11548-020-02236-6)
Supplement: Supplementary file 4 — Online Resource 4: Interview guideline (PDF 277 kb) [file 11548_2020_2236_MOESM4_ESM.pdf]

International Journal of Computer Assisted Radiology and Surgery

Online Resource 4: Interview Guideline

Ultrasound in augmented reality: a mixed-methods evaluation of head-mounted displays in image-guided interventions

Christoph Rüger [1, 3, 5]
rueger@campus.tu-berlin.de

Markus A. Feufel, Prof. Dr. [4]
markus.feufel@tu-berlin.de

Simon Moosburner [1]
simon.moosburner@charite.de

Christopher Özbek, Dr. [3]
coezbek@scopis.com

Johann Pratschke, Prof. Dr. med. [1, 2]
johann.pratschke@charite.de

Igor M. Sauer, Prof. Dr. med. [1, 2]
igor.sauer@charite.de
(corresponding author)

1. Department of Surgery, Campus Charité Mitte | Campus Virchow-Klinikum, Experimental Surgery, Charité – Universitätsmedizin Berlin, corporate member of Freie Universität Berlin, Humboldt-Universität zu Berlin, and Berlin Institute of Health, 13353 Berlin, Germany
2. Cluster of Excellence Matters of Activity. Image Space Material funded by the Deutsche Forschungsgemeinschaft (DFG, German Research Foundation) under Germany´s Excellence Strategy – EXC 2025, Augustenburger Platz 1, 13353 Berlin, Germany
3. Scopis GmbH
   Heinrich-Heine-Platz 10, 10179 Berlin
4. Technische Universität Berlin
   Department of Psychology and Ergonomics, Division of Ergonomics
   Marchstr. 23, MAR 3-2, 10587 Berlin, Germany
5. Technische Universität Berlin
   Straße des 17. Juni 135, 10623 Berlin

International Journal of Computer Assisted Radiology and Surgery

Original version in German – English translation below

Einführung

- Kurze Pause, HL ablegen, Glas Wasser anbieten, „durchatmen"
- Interview ist eher offen – bei der Beantwortung durch Probanden können durchaus „Abschweifer" gemacht werden
- Im Gegensatz zu lautem Denken ist hier Retrospektion und Interpretation des Handelns gefragt!

Physische Ergonomie

- Empfanden Sie die Aufgabe mittels konventionellen Bildschirmes zu irgendeinem Zeitpunkt körperlich unangenehm? Z. B. Belastung des Rückens oder Nackens, angestrengte Augen…
  - Wenn ja: Wie lange würden Sie diese Unannehmlichkeiten während Ihrer regulären Arbeit tolerieren?
  - Wenn ja: Wie stark schätzen Sie den Einfluss auf Ihre Arbeit ein?
- Empfanden Sie die Aufgabe mittels HMD zu irgendeinem Zeitpunkt körperlich unangenehm? Z. B. Belastung des Rückens oder Nackens, angestrengte Augen…
  - Wenn ja: Wie lange würden Sie diese Unannehmlichkeiten während Ihrer regulären Arbeit tolerieren?
  - Wenn ja: Wie stark schätzen Sie den Einfluss auf Ihre Arbeit ein?

Herangehensweise

- Welche Sinneseindrücke benutzen Sie zum Lösen der Aufgabe?
- Hat sich das Tragen des HMD darauf ausgewirkt, wie Sie die Aufgabe lösen?
  - Wenn ja, inwiefern?
  - Wenn ja, wie bewerten Sie diese Wirkung?
- Denken Sie, dass sich, bei höherer Vertrautheit mit der Technik, dies deutlich ändern würde?
- Haben Sie Unterschiede zwischen HMD und konventionellem Bildschirm bezüglich einem oder dem Zusammenspiel mehrerer dieser Sinneseindrücke erlebt?

Limitationen

- Waren Sie bei der Lösung der Aufgabe mittels konventionellen Bildschirmes zu irgendeinem Zeitpunkt genervt, frustriert o. ä.?
- Waren Sie bei der Lösung der Aufgabe mittels HMD zu irgendeinem Zeitpunkt genervt, frustriert o. ä.?
  - Wenn ja, denken Sie, dass diese Probleme durch größere Vertrautheit mit der Technik gemindert werden könnten?
  - Wenn ja, denken Sie, dass diese Probleme durch technische Verbesserungen gemindert werden könnten?

- Haben Sie unter Einsatz des HMD Ihre Ziele erreichen können oder haben Sie sich zu irgendeinem Zeitpunkt technisch limitiert gefühlt?
  - Wenn limitiert: Konnten Sie die Aufgabe trotzdem für Sie zufriedenstellend lösen?
  - Wenn ja, wie haben Sie die Limitationen umgangen?
- Empfanden Sie die Überlagerung der virtuellen Elemente jemals als störend?
- Sehen Sie sonstige Hürden oder Limitationen bezüglich des Einsatzes in der Praxis?

Vorteile

- Waren für Sie ein oder mehrere Vorteile bei der Durchführung mittels HMD im Vergleich zum konventionellen Bildschirm erkenntlich?

- Wenn ja, welche? Wie viel technischer Aufwand wäre durch diese Vorteile gerechtfertigt?
- Könnten diese Vorteile auch durch andere, einfachere technische Maßnahmen erzielt werden?
- Wie würde sich mehr Übung im Einsatz des HMDs auf diese Vorteile auswirken?

Freier Teil

- Sind Ihnen weitere Unterschiede zwischen der Durchführung mit konventionellem Bildschirm und mit HMD aufgefallen?
- Fallen Ihnen potentielle Vorteile der Technik in ähnlichen Einsatzgebieten ein?
- Evtl. auf Äußerungen während des Lauten Denkens einzugehen

Abschließendes Urteil

- Würden Sie diese Technologie in Zukunft gerne während Ihrer Arbeit im OP einsetzen?
  - Wenn ja, in welchem Rahmen und wieso?
  - Wenn nein, aus welchen Gründen?

English Translation

Introduction

- Take off HoloLens, short break, offer water and snacks
- Interview is not strictly bound to questions – going off on a tangent is fine
- In contrast to thinking-aloud, restrospective thoughts and conscious interpretations of what happens are the priority now

Physical ergonomics

- Did you find the task to be physically uncomfortable at any point when using the conventional monitor? For example straining your neck, back or eyes…
    - If so: For how long would you want to tolerate such discomforts during your regular work?
    - If so: How much do you reckon does it impact your work?
- Did you find the task to be physically uncomfortable at any point when using the AR-HMD? For example straining your neck, back or eyes…
    - If so: For how long would you want to tolerate such discomforts during your regular work?
    - If so: How much do you reckon does it impact your work?

Approach to solving the task

- Which sensory input did you use to solve the task?
- Did the HMD change how you completed the task?
    - If so, how?
    - If so, how do you view this change?
- Do you think that this would change if you were more familiar with the HMD?
- Did you notice differences regarding your perception or the interaction between multiple sensory inputs when wearing the HMD, compared to the conventional monitor?

Limitations

- When working on the task, did you at any point feel frustrated, annoyed etc. when using the conventional monitor?
- When working on the task, did you at any point feel frustrated, annoyed etc. when using the conventional monitor?
    - If so, do you think these issues may be reduced or mitigated through practice?
    - If so, do you think these issues may be reduced or mitigated through technical improvements?

- Could you reach your goals when using the HMD without issues or did you at any point feel technologically limited?
    - If limited: Could you still solve the task to your satisfaction?
    - If so, how did you work around the limitations?
- Did you ever find the overlay of virtual elements to be distracting or limiting?
- Do you see any other potential hurdles or limitations regarding the practical use of this technology?

Benefits

- Did you find any benefits in using the AR-HMD, compared to the conventional monitor?
    - If so, which? How much technical complexity would these benefits justify?
- Do you think these benefits could also be achieved through easier technological means?
- Which effects would more practice with the AR-HMD have on these benefits?

Open questions

- Did you notice any other differences between using the AR-HMD and the conventional monitor?
- Do you see any potential benefits in similar use-cases?
- (Potentially talk about statements from thinking-aloud part of experiment)

Final verdict

- Would you like to use this technology in a professional context in the future?
    - If so, in which capacity and for which reasons?
    - If not, for which reasons?
