# Supplementary material for: Ultrasound in augmented reality: a mixed-methods evaluation of head-mounted displays in image-guided interventions
Source: Int J Comput Assist Radiol Surg. 2020 Jul 28;15(11):1895–905. doi: 10.1007/s11548-020-02236-6 (PMC8332636; doi:10.1007/s11548-020-02236-6)
Supplement: Supplementary file 5 — Online Resource 5: Category definition (categories of content analysis) (PDF 109 kb) [file 11548_2020_2236_MOESM5_ESM.pdf]

International Journal of Computer Assisted Radiology and Surgery

Online Resource 5: Category definition (categories of content analysis)

Ultrasound in augmented reality: a mixed-methods evaluation of head-mounted displays in image-guided interventions

Christoph Rüger [1, 3, 5]
rueger@campus.tu-berlin.de

Markus A. Feufel, Prof. Dr. [4]
markus.feufel@tu-berlin.de

Simon Moosburner [1]
simon.moosburner@charite.de

Christopher Özbek, Dr. [3]
coezbek@scopis.com

Johann Pratschke, Prof. Dr. med. [1, 2]
johann.pratschke@charite.de

Igor M. Sauer, Prof. Dr. med. [1, 2]
igor.sauer@charite.de
(corresponding author)

1. Department of Surgery, Campus Charité Mitte | Campus Virchow-Klinikum, Experimental Surgery, Charité – Universitätsmedizin Berlin, corporate member of Freie Universität Berlin, Humboldt-Universität zu Berlin, and Berlin Institute of Health, 13353 Berlin, Germany
2. Cluster of Excellence Matters of Activity. Image Space Material funded by the Deutsche Forschungsgemeinschaft (DFG, German Research Foundation) under Germany´s Excellence Strategy – EXC 2025, Augustenburger Platz 1, 13353 Berlin, Germany
3. Scopis GmbH
Heinrich-Heine-Platz 10, 10179 Berlin
4. Technische Universität Berlin
Department of Psychology and Ergonomics, Division of Ergonomics
Marchstr. 23, MAR 3-2, 10587 Berlin, Germany
5. Technische Universität Berlin
Straße des 17. Juni 135, 10623 Berlin

Original version in German – English translation below

Categories are denoted as letters (N, L, …), subcategories as digits following the category letter. The first subcategory of each category defines the general category and is used when a phrase belongs to the category, but not to any of the defined subcategories.

Participants are referred to as P and a number, for example P2 or P4.

	**Inhaltliche Beschreibung**	**Beispiele für Anwendung**	**Abgrenzungen**
N1: Nutzen	Wird kodiert, wenn ein konkret bzw. direkt erlebter/ wahrgenommener Nutzen beschrieben wird		Spekulative bzw. vorhergesagte Nutzen (z. B. bei anderen Anwendungsfällen und für andere Personen) werden als *Einschätzung* kodiert, ebenso unspezifisch positive Aussagen
N2: Orientierung erleichtert	Das HMD erleichtert die Verortung des Ultraschallbildes in der Anatomie und/oder des Schallkopfes auf dem Patienten	„[…] macht sozusagen die Verortung von dem Ultraschallbild im Körper deutlich einfacher.“ (P4)<br><br>„also es war mit der Brille deutlich einfacher, visuell nochmal zu kontrollieren, […], wo der Schallkopf sich am Patient befindet“ (P3)	In dieser Kategorie stehen einzelne Schnittbilder im Vordergrund, beim Zusammenwirken mehrerer Bilder oder räumlicher Korrelation ist N3 zu kodieren
N3: Verbesserte 3D-Vorstellung	Das HMD erleichtert die 3D-Vorstellung anatomischer Strukturen und der Korrelation mehrerer Bilder untereinander	„das ist schon ein gewisses 3D-Gefühl für den Körper, das auf dem Schallbildschirm ja immer nur sozusagen virtuell rekonstruiert wird“ (P3)	
N4: Hedonistische Aspekte	Subjektive Ausdrücke von Spaß, Begeisterung und/oder bezüglich des Novums der Technologie	„Also macht Bock halt“ (P1, S.4)<br>„Das ist schon abgefahren“ (P1, S.2)<br>„[…], dass es halt irgendwie so was Neues ist, was ja auch irgendwie total spannend ist.“ (P2)	Eher analytische Aussagen wie „das ist sinnvoll“ werden als *Einschätzung* kodiert
L1: Limitation	Wird kodiert, wenn eine Limitation des HMDs bezüglich der Benutzung im Kontext der Sonographie beschrieben wird		Unspezifische negative Urteile, wie „das ist nicht sinnvoll“, werden als Einschätzung kodiert
L2: Blickwinkel	Die Sichtbarkeit oder Qualität des Ultraschallbildes im HMD wird durch die perspektivische Darstellung gemindert	„Aber wenn ich jetzt hierhin gucke, sehe ich wenig, weil das Bild jetzt ganz, ganz in meinem so Blick ist“ (P1)	
L3: Ergonomie	Aspekte des technischen Aufbaus mit HMD schränken den physischen Komfort des Benutzenden ein	„[…] ich fand's ein bisschen schwer auf dem Kopf.“ (P4)	Bemerkungen, die spezifisch auf die erforderliche Bewegung hinweisen, gehören zu Kategorie *L5*
L4: Haptische Bedienung	Die Betätigung der haptischen Bedienelemente des Ultraschallgeräts ist mit HMD schwieriger	„es war aber schwieriger nebenher dann aber das Gerät zu bedienen […]“ (P3)	
L5: Bewegung	Die Darstellung im HMD	„das ist gerade ein bisschen	Wird nicht kodiert, wenn

	Inhaltliche Beschreibung	Beispiele für Anwendung	Abgrenzungen
erforderlich	erfordert ein höheres Maß an Bewegung des Benutzenden	anstrengend, dass man dann den Kopf so dreht, und dann wirklich immer so auf der Ebene der Ultraschallsonde ist“ (P5)	die Bewegung nicht ausgeführt wird/wurde („ich müsste mich bewegen“), dieser Fall gehört zu L2
L6: Einstellung mit Brille nicht/schwer möglich	Gewünschte Schnittbilder/-ebenen können nicht mit dem HMD dar-/eingestellt werden	„Ich kann hier beim besten Willen nicht mit der Brille das einstellen.“ (P2)	
L7: Optisches Tracking	Der erforderliche Blickkontakt der Sonde zur 3D-Kamera limitiert die Untersuchung	„aber wenn ich jetzt versuche die Punkte so zur 3D-Kamera zu halten kann ich dann auf [jetzigem "Testpatient"] nichts mehr sehen.“ (P1)	Falls sowohl Limitationen durch das Tracking *als auch* durch die Blickwinkelabhängigkeit relevant sind, ist L7 zu kodieren.
L8: Bildqualität	Auflösung, Kontrast oder Latenz des Bildes im HMD fallen störend auf	„Es wirkt alles so ein bisschen zeitversetzt gerade, also vom Gefühl.“ (P5, S. 2)<br>„ich muss sagen die Bilder waren für mich halt viel klarer. [Auf dem konventionellen Bildschirm]“ (P6)	
G1: Gewöhnung	Kodiert, wenn Äußerungen eine ungewohnte oder verwirrend Situation, aber auch das Überwinden dieser beschreibt		
G2: Ungewohnt	Äußerungen, die konkret Ungewohntheit ausdrücken	„ist ein bisschen ungewohnt so zu schauen“ (P3)	
G3: Adaptation	Wird kodiert bei Verbalisierung der Bewältigung einer ungewohnten oder verwirrende Situation	„Hm. So. Leber. So. Jetzt wird's besser.“ (P1)<br>„Mittlerweile gewöhnt man sich dran“ (P5)	
G4: Verwirrung	Äußerungen, die konkret Verwirrung und/oder, auch implizit, fehlendes Verständnis der Anwendung ausdrücken, auch sprachlich durch unvollständige Sätze bemerkbar	„Okay, ähm. Ich muss mir das noch einen Moment angucken, um zu wissen was ich sehe“ (P6)<br>„Hm. Klappt das hier? Vielleicht muss man das irgendwie anders machen mit der...“ (P1)	
G5: Übung bringt Besserung	Vorhersage, dass Verwirrung oder Limitationen durch das HMD durch Übung/ Wiederholung reduziert oder eliminiert werden kann	„Also ich glaub es wird besser, wenn man sich dran gewöhnt, […]“ (P5)<br>„Also ich denke, wenn ich da eingewöhnt wär, könnte ich das auch deutlich besser anwenden.“ (P6)	Wird nur kodiert, wenn die Aussage auch selbstbezogen ist, ansonsten als *Einschätzung*
E1: Einschätzung	Aussagen zum Einsatz bzw. zu Auswirkungen des HMDs mit Sonographie, die sich nicht direkt auf den im Experiment erlebten Anwendungsfall beziehen bzw. sich davon nicht direkt ableiten		
E2: Für Punktionen sinnvoll	Einsatz erscheint für Punktionen sinnvoll	„Im klinischen Alltag für Biopsien fände ich das jetzt recht sinnvoll.“ (P3)	
E3: Intraoperativ sinnvoll	Einsatz erscheint intraoperativ sinnvoll	„Also bei Leberresektionen da draufzuschallen direkt, um da zu gucken, wo ist mein Knoten, das ist schon spannend.“ (P1)	
E4: Für Lehre sinnvoll	Einsatz erscheint in Lehre/im Unterricht sinnvoll	„Also für die Lehre finde ich's deutlich sinnvoller, dass, äh, die Studenten die Schnittansicht verstehen,“ (P3)	

	**Inhaltliche Beschreibung**	**Beispiele für Anwendung**	**Abgrenzungen**
E5: Für multimodalen Vergleich sinnvoll	Einsatz könnte im Vergleich von Ultraschallbildern mit 3D-Daten sinnvoll sein	„Ich denke eher für so spezielle Sachen, wo man Sachen korrelieren muss mit anderen Sachen, die man dann schon sieht, also intermodal, intermodaler Bildvergleich und so.“ (P5)	
E5: In Zukunft gerne benutzen	Proband_in würde HMD-gestützte Sonographie, auch unter Einschränkungen, gerne in Zukunft nutzen	„Also ich denke, wenn ich sie besser einsetzen könnte, würde ich es auf jeden Fall probieren.“ (P6)	
M1: Machbarkeit	Aussagen, welche möglicherweise limitierende Faktoren als positiv oder tolerabel bezeichnen		
M2: Bildqualität	Ausreichende oder gute Bildqualität und -latenz	„Und die Bildqualität ist eigentlich auch ziemlich gut, muss man sagen.“ (P4)	
M3: Ergonomie	Ausreichende bzw. tolerierbare ergonomische Eigenschaften des Systems	„Ich steh ja auch mal 6 Stunden im OP, also Körperhaltung wäre jetzt nicht so das Problem, wenn es da mal zwackt.“ (P6)	
W1: Wahrnehmung	Aussagen bezüglich der verwendeten Sinne und deren Zusammenspiel		
W2: Wahrnehmungsart	Bezeichnung oder Nennung eines verwendeten Wahrnehmungssinnes	„Ich würde mal sagen 98% visuell, der Rest haptisch?“ (P6)	
W3: Kein Unterschied	Kein Unterschied in Wahrnehmung bzw. dem Zusammenspiel der Sinneseidrücke zwischen HMD und konventionellem Monitor	„[Zusammenspiel der Sinneseindrücke] Würde ich als relativ ähnlich bezeichnen bei beiden.“ (P4)	
W4: Unterbewusste Propriozeption	Die Wahrnehmung und Steuerung der Bewegung der Sonde erfolgt unterbewusst	„[…] ich guck selten wirklich auf den, also während der Untersuchung irgendwo noch auf den Patienten sondern lass mich da halt vom Gefühl leiten.“ (P2)	

English Translation

Categories are denoted as letters (N, L, …), subcategories as digits following the category letter. The first subcategory of each category defines the general category and is used when a phrase belongs to the category, but not to any of the defined subcategories.

Participants are referred to as P and a number, for example P2 or P4.

	**Description**	**Examples**	**Differentiations to similar categories**
N1: Benefits	Coded when a concrete or directly experienced/perceived benefit is described		Speculative or predicted benefits (e.g. for other applications and for other users) are coded as assessments, as are unspecific positive statements
N2: Navigation facilitated	HMD facilitates the localization of the ultrasound image in the anatomy and/or the transducer on the patient	„[…] makes it much easier to locate the ultrasound image in the body.“ (P4)<br><br>„so it was much easier with the glasses to visually confirm [...] where the transducer is located on the patient“ (P3)	For this category, only the effect on individual ultrasound images is relevant; if several images are combined or spatial correlation is required, N3 is to be coded
N3: Improved 3D understanding	HMD facilitates 3D understanding of anatomical structures and the correlation of several images	„this is actually kind of a 3D-feeling, which otherwise is only virtually reconstructed with a monitor, basically“ (P3)	
N4: Hedonistic aspects	Phrases expressing enjoyment, amazement or novelty value of technology	„So it‘s definitely fun“ (P1)<br><br>„That really is cool“ (P1)<br><br>„[…] that it‘s something new somehow, which is super interesting in a way, too..“ (P2)	Analytical statements such as ‚I think it‘s useful‘ are to be coded as *Assessments*
L1: Limitations	Is coded if participants mention a limitation of the HMD regarding this use case		Unspecific negative statements such as ‚I don‘t think it‘s useful‘ are to be coded as *Assessments*
L2: Angle of view	Visibility or quality of the ultrasound image (viewed through the HMD) is limited due to the perspective rendering of the image	„But if I look there I see hardly anything because the image is kind of along my view now“ (P1)	
L3: Ergonomics	Aspects of technical estup with HMD decrease physical comfort of participant	„[…] I thought it was kind of heavy.“ (P4)	Statements which specifically point to the need for additional movement belong to category *L5*

	**Description**	**Examples**	**Differentiations to similar categories**
L4: Haptical input	Using haptic input elements of the ultrasound system is harder when using the HMD	„but it was more difficult then to operate the system on the side […]" (P3)	
L5: Movement required	Visualization through the HMD requires users to move to see some views	„that's a bit exhausting right now, that you have to rotate your head like this so that you're always really aligned to the plane of the ultrasoun image" (P5)	Not coded if movement was not executed („I would need to move") - this rather belongs to cartegory L2
L6: Finding views with HMD is difficult	Certain ultrasound views/planes cannot be appropriately viewed with the HMD	„I really tried but I can't get this with the HMD." (P2)	
L7: Optical Tracking	The required line of sight between the trackers and the 3D camera limits the user in performing the task	„but if I try to point the spheres to the 3D camera like this, I can't see anything on the patient anymore " (P1)	If limitations occur due to *both* tracking and angle of view, code L7
L8: Image quality	Resolution, contrast or latency of the image are insufficient	„things feel a little delayed right now." (P5)<br>„I have to say that the images were a lot clearer there. [on conventional ultrasound monitor]" (P6)	
G1: Unfamiliarity and adaption	Coded if statements describe an unfamiliar or confusing situation, but also if such a situation is overcome		
G2: Unfamiliar	Statements directly mentioning unfamiliarity	„it's a little unfamiliar to see it like this" (P3)	
G3: Adaption	Coded if participants verbalize overcoming a confusing or unfamiliar situation	„Hm. Right. Liver. Okay. Getting better now." (P1, S. 2)<br>„Starting to get used to it by now" (P5)	
G4: Confusion	Statements which directly mention confusiong and/or implicity express lack of understanding of the HMD application	„Okay, uh. I need to keep looking at this for a bit so I know what I'm seeing" (P6)<br>„Hm. Does that work? Maybe I need to do something differently with the..." (P1)	
G5: Improvements through practice	Prediction that confusion or limitations caused by the HMD can be reduced or eliminated through practice	„Well I think it will get better once I get used to it […]" (P5)<br>„I think if I was familiar with it, I could utilize it a lot better." (P6)	Only to be coded if participants refer to themselves, otherwise code as *Assessment*
E1: Assessments	Statements regarding effects of HMDs in this use-case, which are not directly related to the experiment or cannot be directly derived from it		
E2: Useful for needle placements	Deemed as useful for needle placements	„In clinical practice I'd find this useful for biopsies." (P3, S. 4)	
E3: Useful for surgery	Deemed as useful for intraoperative use	„For liver resections with ultrasound, to check where a tumor is, that would definitely be	

	Description	Examples	Differentiations to similar categories
		exciting.“ (P1)	
E4: Useful for teaching	Deemed as useful for teaching medical students or junior physicians	„So I think it makes a lot more sense for teaching so that students can better understand ultrasound as sectional images“ (P3)	
E5: Useful for multi-modal image comparison	Deemed as useful for copmaring ultrasound images with other 3D data	„I‘m rather thinking kinda special stuff, where you need to correlate some things with others, so intermodal, intermodal image comparison and stuff like that.“ (P5)	
E5: Would like to use HMDs in the future	Participants would like to test HMDs in practice in the future, even with limitations	„So I think if I could use it better, I would definitely want to try.“ (P6)	
M1: Feasibility	Statements which indicate that potentially limiting factors are acceptable or good		
M2: Image quality	Sufficient or good image quality and latency	„And the quality of the image is quite good, too, I have to say.“ (P4)	
M3: Ergonomics	Sufficient or tolerable ergonomic properties of HMD	„I stand in the OR for 6 hours straight occasionally, so body posture wouldn‘t be the issue if it‘s a little uncomfortable here and there“ (P6)	
W1: Perception	Statements regarding sensory perceptions used by participants to complete the task		
W2: Perception type	Naming of specific perception types	„I‘d say 98% visually, otherwise haptics?“ (P6)	
W3: No difference	No differences in perception or the interaction of senses between using the HMD and the conventional monitor	„[Interactions between senses] I‘d describe that as quite similar for both.“ (P4)	
W4: Subconscious proprioception	Perception and control of the ultrasound prove is subconscious	„[…] I hardly ever really look at neither the probe nor anywhere on the patient, I‘m simply following my feelings there.“ (P2)	
