# Supplementary material for: Ultrasound in augmented reality: a mixed-methods evaluation of head-mounted displays in image-guided interventions
Source: Int J Comput Assist Radiol Surg. 2020 Jul 28;15(11):1895–905. doi: 10.1007/s11548-020-02236-6 (PMC8332636; doi:10.1007/s11548-020-02236-6)
Supplement: Supplementary file 7 — Online Resource 7: Quantitative Data (PDF 667 kb) [file 11548_2020_2236_MOESM7_ESM.pdf]

International Journal of Computer Assisted Radiology and Surgery

Online Resource 7: Quantitative Data

Ultrasound in augmented reality: a mixed-methods evaluation of head-mounted displays in image-guided interventions

Christoph Rüger [1, 3, 5]
rueger@campus.tu-berlin.de

Markus A. Feufel, Prof. Dr. [4]
markus.feufel@tu-berlin.de

Simon Moosburner [1]
simon.moosburner@charite.de

Christopher Özbek, Dr. [3]
coezbek@scopis.com

Johann Pratschke, Prof. Dr. med. [1, 2]
johann.pratschke@charite.de

Igor M. Sauer, Prof. Dr. med. [1, 2]
igor.sauer@charite.de
(corresponding author)

1. Department of Surgery, Campus Charité Mitte | Campus Virchow-Klinikum, Experimental Surgery, Charité – Universitätsmedizin Berlin, corporate member of Freie Universität Berlin, Humboldt-Universität zu Berlin, and Berlin Institute of Health, 13353 Berlin, Germany
2. Cluster of Excellence Matters of Activity. Image Space Material funded by the Deutsche Forschungsgemeinschaft (DFG, German Research Foundation) under Germany´s Excellence Strategy – EXC 2025, Augustenburger Platz 1, 13353 Berlin, Germany
3. Scopis GmbH
   Heinrich-Heine-Platz 10, 10179 Berlin
4. Technische Universität Berlin
   Department of Psychology and Ergonomics, Division of Ergonomics
   Marchstr. 23, MAR 3-2, 10587 Berlin, Germany
5. Technische Universität Berlin
   Straße des 17. Juni 135, 10623 Berlin

Please note that this data is also available in *.csv* format at
http://dx.doi.org/10.14279/depositonce-9478

Conventional (without HMD)							With HMD							Differences			Participant Data		
Placement Error [mm]			Task Duration [s]			Task Load	Placement Error [mm]			Task Duration [s]			Task Load	Placement Error [mm]	Task Duration [s]	Task Load	Started with HMD	Ultrasound examinations performed	Gender
1st try	2nd try	**Mean**	1st try	2nd try	**Mean**	**TLX**	1st try	2nd try	**Mean**	1st try	2nd try	**Mean**	**TLX**						
19,5	15,2	17,35	163	154	158,5	84,00	4,3	1,1	2,7	100	119	109,5	76,67	-14,65	-49,00	-7,33	FALSE	5	f
5,1	1,7	3,4	38	19	28,5	39,00	2,1	4,4	3,25	23	23	23	52,33	-0,15	-5,50	13,33	FALSE	15	m
6,4	0,9	3,65	58	63	60,5	19,00	4,9	4,8	4,85	49	43	46	41,00	1,20	-14,50	22,00	TRUE	500	m
11,6	4,5	8,05	93	75	84	49,30	1,6	2,1	1,85	97	69	83	57,33	-6,20	-1,00	8,03	TRUE	40	m
9,5	15,6	12,55	55	27	41	49,30	5,7	4,2	4,95	21	30	25,5	48,00	-7,60	-15,50	-1,30	FALSE	150	m
3,2	3,1	3,15	32	32	32	51,33	3	3,8	3,4	56	53	54,5	70,67	0,25	22,50	19,33	TRUE	200	m
0,7	7,1	3,9	113	46	79,5	26,67	11,3	4,3	7,8	76	104	90	56,67	3,90	10,50	30,00	FALSE	3	f
2,4	4,2	3,3	38	53	45,5	27,00	4	2	3	35	38	36,5	40,67	-0,30	-9,00	13,67	FALSE	8	m
18,3	16,3	17,3	63	67	65	41,00	10,3	4,2	7,25	27	85	56	59,00	-10,05	-9,00	18,00	TRUE	30	m
8,7	6,3	7,5	51	42	46,5	42,67	2,6	0,1	1,35	45	41	43	39,00	-6,15	-3,50	-3,67	TRUE	450	m
9,6	1,3	5,45	15	247	131	55,00	10,1	5,1	7,6	199	103	151	69,33	2,15	20,00	14,33	TRUE	10	f
13,1	4,1	8,6	139	149	144	47,67	2,6	1,5	2,05	99	98	98,5	41,67	-6,55	-45,50	-6,00	FALSE	30	m
11,1	11,3	11,2	63	86	74,5	52,67	11,8	8,1	9,95	132	141	136,5	62,00	-1,25	62,00	9,33	TRUE	12	f
5,3	2,6	3,95	37	27	32	18,00	2,3	10,2	6,25	53	57	55	44,00	2,30	23,00	26,00	TRUE	50	m
6,3	11,2	8,75	285	125	205	52,00	8,2	14,2	11,2	118	150	134	51,00	2,45	-71,00	-1,00	FALSE	4	f
12,9	2,8	7,85	65	65	65	66,67	11,5	1,3	6,4	92	68	80	39,33	-1,45	15,00	-27,33	TRUE	18	m
5,7	0,9	3,3	182	11	96,5	34,00	2,8	2,3	2,55	14	15	14,5	24,33	-0,75	-82,00	-9,67	FALSE	400	f
8,4	4,3	6,35	63	54	58,5	47,33	2,3	5,4	3,85	37	60	48,5	27,33	-2,50	-10,00	-20,00	FALSE	650	m
5,5	3,7	4,6	26	14	20	25,67	3,6	0,4	2	22	21	21,5	29,00	-2,60	1,50	3,33	TRUE	160	f
9,2	7,6	8,4	19	23	21	41,33	9,4	4,3	6,85	26	15	20,5	42,67	-1,55	-0,50	1,33	TRUE	250	m
